# Effect of phenotypic detection of circulating tumor cells marked by epithelial-mesenchymal transformation on the prognosis of lung cancer: A protocol for systematic review and meta-analysis

**DOI:** 10.1097/MD.0000000000022960

**Published:** 2020-10-30

**Authors:** Bin Ge, Yong Wang, Shaoqing Lei, Jincao Zhang

**Affiliations:** a Department of laboratory medicine, People's Hospital of Qichun County, Hubei Province; b Department of anesthesiology, Hubei Provincial People's Hospital; c Department of laboratory medicine, Yingcheng City People's Hospital, Hubei Province, Hubei, China.

**Keywords:** circulating tumor cells, epithelial-mesenchymal transformation, lung cancer, meta-analysis, prognosis, protocol, systematic review

## Abstract

**Background:**

To explore the significance of phenotype detection of circulating tumor cells (CTCs) based on epithelial-mesenchymal transition (EMT) labeling to evaluate the prognosis of lung cancer.

**Methods:**

Database was retrieved from China National Knowledge Infrastructure (CNKI), Chinese Biomedical literature Database (CBM), Chinese Scientific and Journal Database (VIP), Wan Fang database, PubMed, and EMBASE. Based on EMT on overall survival (OS) and disease-free survival (DFS), hazard ratios (HRs) and its 95% of confidence intervals (CIs) were applied to assess the prognostic effect of CTCs. RevMan 5.3 and STATA 16.0 software were adopted to perform the meta-analysis.

**Results:**

Based on EMT in terms of the prognosis of patients suffering from lung cancer, this study comprehensively reviewed and evaluated the available evidence of phenotype detection of CTCs.

**Conclusion:**

Based on EMT in the prognosis of patients who developed with lung cancer, our findings proved the effect of phenotype detection of CTCs. Such studies may reveal a new prognostic marker for lung cancer patients and help clinicians and health professionals make clinical decisions.

**OSF Registration Number:**

DOI 10.17605/OSF.IO/E7KAZ.

## 1 Introduction

According to the statistics conducted by World Health Organization (WHO), the overall morbidity and mortality of lung cancer ranks first in the world,^[1]^ and its incidence maintains a rising trend year by year. The incidence of lung cancer is always the highest among men and second only to breast cancer in women. Although, in recent years, surgery, radiotherapy, chemotherapy and molecular targeted therapy have greatly improved the treatment of lung cancer, the 5-year overall survival rate is still low, so lung cancer is a major disease threatening people's health.^[2,3]^

Invasion and metastasis of lung cancer is the main reason that affects the quality of patients life, thus leading to poor therapeutic effect.^[2]^ The lack of sensitive and effective methods for the detection of early invasion and metastasis of lung cancer is the main reason for the failure of detecting early lung cancer and intervention, and it is also an important factor for the increase of mortality.^[4]^

At present, it is believed that circulating tumor cells (CTCs) invade the blood from the site of the primary tumor, and is the “seed” of metastasis.^[5]^ CTCs have gradually become a “real-time tumor liquid biopsy” marker for the monitoring of metastasis, and the prognosis and curative effect evaluation of lung cancer. However, due to the loss of some epithelial markers of tumor cells that are caused by epithelial-mesenchymal transition (EMT), in the process of tumorigenesis and development, the widely used detection method of epithelial cell markers has obvious defects. EMT is considered as one of the key mechanisms of invasion and metastasis of lung cancer.^[6]^ Therefore, the detection and analysis of interstitial CTCs in lung cancer is of great value clinically.

At present, the relationship between the phenotypic detection of CTCs based on EMT and the clinicopathological features and prognosis of lung cancer is still controversial. This meta-analysis would further explore the relationship between the detection of CTC phenotype based on EMT and the clinicopathological features and prognosis of lung cancer.

## 2 Methods

### 2.1 Study registration

This meta-analysis protocol is based on the statement guidelines of Preferred Reporting Items for Systematic Reviews and Meta-analysis Protocols (PRISMA-P).^[7]^ The PRISMA-P checklist for the protocol is provided in the PRISMAP-checklist. The protocol of the systematic review has been registered on Open Science Framework, and the registration number is DOI 10.17605/OSF.IO/E7KAZ.

### 2.2 Data sources and search strategy

We searched China National Knowledge Infrastructure (CNKI), Chinese Biomedical literature Database (CBM), Chinese Scientific and Journal Database (VIP), Wan Fang database, PubMed, and EMBASE without language restrictions. The detailed PubMed search strategy is illustrated in Table 1. Similar search strategies are adopted for the retrieval of other electronic databases.

**Table 1:**
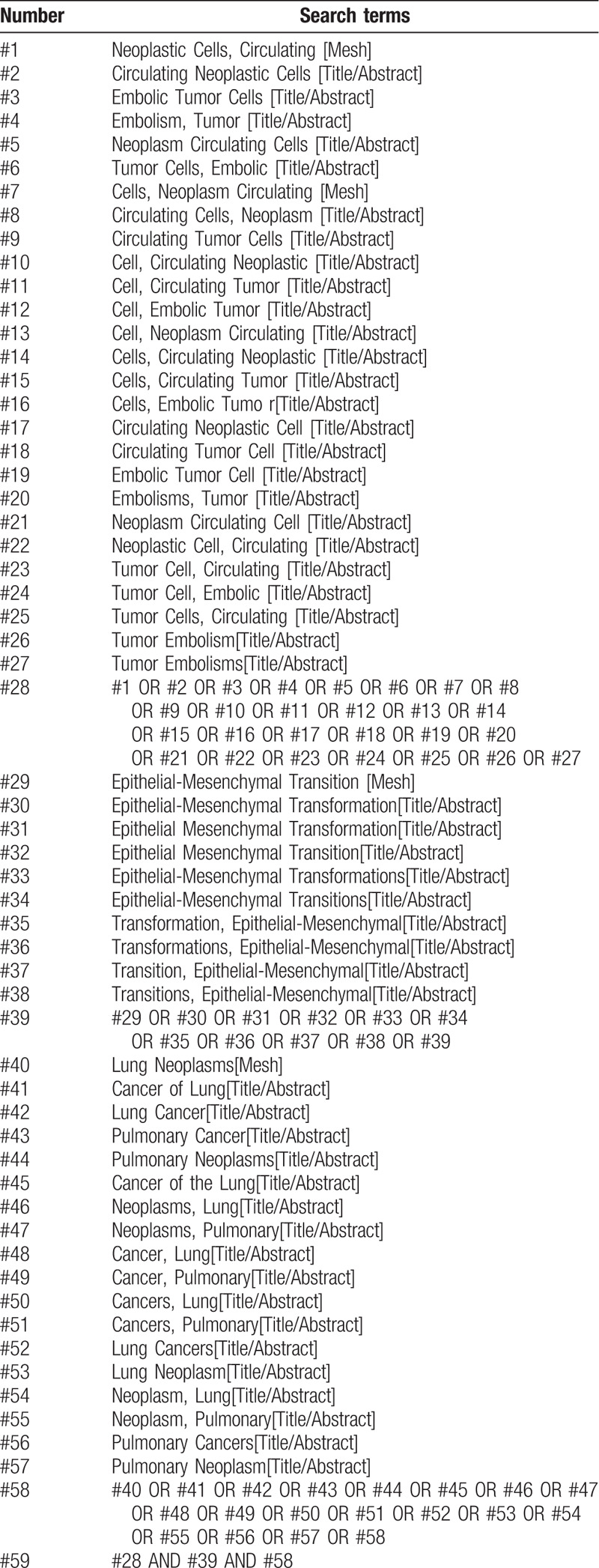
Search strategy in PubMed database.

### 2.3 Inclusion criteria for study selection

1. Lung cancer patients based on pathology and histology.
2. Reported survival-related data, including overall survival (OS) and disease-free survival (DFS).
3. According to the EMT typing and counting of CTCs, it can be divided into mixed type and non-mixed type, and Patients are divided into mixed type and non-mixed type.
4. The article proved the relationship between the types of CTCs based on EMT and clinical pathological characteristics.
5. Published full-text articles, and original Chinese and English Research papers.

If there are repeated articles, the 1 with higher quality and larger sample size is chosen. Conference abstract, case series reports, letters, animal experiments, land lack of measurement indicators and survival research are not included.

### 2.4 Data collection and analysis

#### 2.4.1 Selection of studies

**Figure 1:**
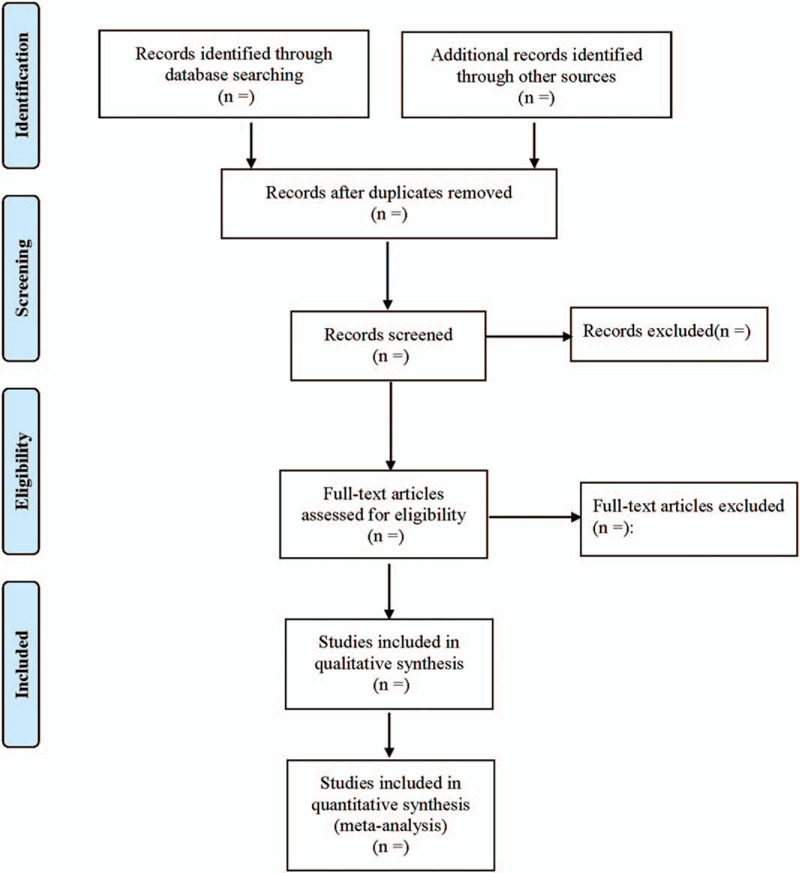
Flow diagram of study selection process.

The 2 authors independently reviewed the titles/abstracts of all confirmed documents, and all irrelevant studies were excluded. Subsequently, the full text of potentially relevant papers was obtained to determine whether they meet all the inclusion criteria. Any differences would be resolved by consensus with the help of another experienced author. All excluded studies with detailed reasons were recorded at different stages. The flow chart of the research selection (Fig. 1) displays a detailed description.

#### 2.4.2 Data extraction and management

The 2 authors independently collected information from each included study. Any conflicts would be resolved by consensus with the help of a third experienced author. The extracted information includes manuscript name, first author name, journal, publication year, country, race, age, gender, sample size, CTCs detection method, hazard ratios (HRs) and 95% of confidence intervals (CIs) of OS and DFS. We obtained HRs and 95% of CIs from the Kaplan–Meier survival curves by using Engauge Digitizer version 4.1 (http://digitizer.sourceforge.net/). We contacted the lead author to obtain any missing or ambiguities from the included studies information.

### 2.5 Assessment of quality in included studies

The quality assessment of all included studies was conducted independently by 3 researchers. As an observational research bias risk assessment tool recommended by Cochrane Collaboration, Newcastle-Ottawa Quality Assessment Scale (NOS) was used to evaluate the quality of the included studies.^[8]^ All disputes are resolved through discussion. NOS consists of the following 3 quality parameters: selection, comparability and result evaluation. According to these parameters, each study was scored from 0 to 9.^[9–12]^

### 2.6 Measures of prognosis

OS and DFS were taken as prognostic outcomes. The results were expressed as HRs with 95% of CIs.

### 2.7 Management of missing data

When there were insufficient or missing data in the literature, we would contact the author via email, so as to request data. When the data was not available, we only analyzed the currently available data and discussed its potential value.

### 2.8 Statistical analysis

A total of 95% of CIs and HRs were applied to evaluate the relationship between types of CTCs based on EMT with OS and DFS. Statistical heterogeneity tests were performed in the included studies. If there exist no statistical heterogeneity among included literatures (*I*^2^ ≤ 50%, *P* < .1), a fixed effect model is used. When there is statistical heterogeneity among included literatures (*P* < .1, *I*^*2*^ ≥ 50%), the sources of heterogeneity would be analyzed. Clinical heterogeneity was treated by subgroup analysis. In the absence of significant clinical heterogeneity and methodological heterogeneity, statistical heterogeneity was considered, and a random effects model was utilized for analysis. If the clinical heterogeneity of the subgroup analysis is significantly higher, no meta-analysis would be performed and only a descriptive analysis was carried out. Statistical analysis was performed by applying STATA 14.0 (STATA Corporation, College Station, TX, USA) and RevMan 5.3 (The Nordic Cochrane Centre, The Cochrane Collaboration, 2014).

### 2.9 Additional analysis

#### 2.9.1 Subgroup analysis

Based on race, survival data source, and threshold, we conducted subgroup analysis.

#### 2.9.2 Sensitivity analysis

We adopted the one-by-one elimination method to analyze the sensitivity of each indicator, so as to test the stability of the meta-analysis results.

#### 2.9.3 Reporting bias

If the included study ≥10, the funnel plot was used to qualitatively detect publication bias.^[13]^ Besides, Eggers and Beggs test were used for the evaluation of potential publication bias.

## 3 Ethics

Ethical approval is not required, because there is no patient recruitment or personal information collection, and the included data in our study was extracted from published literatures.

## 4 Discussion

Tumor metastasis involves many biological processes, among which the main biological process is that cancer cells enter the blood circulation through the basement membrane to form CTCs, and invade and colonize other tissues and organs.^[14]^ CTCs are closely related to micrometastasis of lung cancer, which is the premise of distant metastasis and the key link in the formation of metastatic foci.^[3,15,16]^

However, the positive rate of CTCs is low. In advanced lung cancer, it is only about 30%, the critical value of CTCs is 1 and 2.^[17]^ The occurrence of EMT in lung cancer makes the existing CTCs detection methods based on epithelial cell markers miss some EMT tumor cells, so the CTCs count is still very low even in patients suffering from advanced lung cancer. The CTCs detection technology only depends on a class of markers obviously and cannot meet the actual needs, so there is an urgent need for new methods based on different phenotypes, including epithelial type, interstitial type, etc.

The EMT of CTCs is a dynamic process, which is dominated by epidermal type in the early stage, mixed type in the middle stage and interstitial type in the late stage.^[18]^ Studies demonstrated that the complete CTCs of EMT has stronger invasive ability and is easier to form metastasis.^[19,20]^ It is closely related to tumor metastasis,^[20]^ because EMT plays an important role in the migration, invasion and formation of metastatic foci of tumor cells. Therefore, the detection of CTCs-EMT phenotype is important in judging tumor prognosis, thus predicting recurrence and metastasis, and evaluating curative effect.

The results of this study could provide the latest evidence for the relationship between EMT-based CTC phenotype detection and clinicopathological features and prognosis of lung cancer, help to establish a feasible choice for clinicians and patients, and offer reliable reference for further research.

## Author contributions

**Conceptualization:** Jincao Zhang, Bin Ge.

**Data curation:** Yong Wang.

**Funding acquisition:** Shaoqing Lei.

**Resources:** Jincao Zhang, Bin Ge.

**Software:** Yong Wang.

**Supervision:** Shaoqing Lei.

**Writing – original draft:** Jincao Zhang, Bin Ge.

**Writing – review & editing:** Jincao Zhang, Bin Ge.
